# Impact of severe secondary tricuspid regurgitation on rest and exercise hemodynamics of patients with heart failure and a preserved left ventricular ejection fraction

**DOI:** 10.3389/fcvm.2023.1061118

**Published:** 2023-03-01

**Authors:** Claudia Baratto, Sergio Caravita, Giorgia Corbetta, Davide Soranna, Antonella Zambon, Céline Dewachter, Mara Gavazzoni, Francesca Heilbron, Michele Tomaselli, Noela Radu, Francesco Paolo Perelli, Giovanni Battista Perego, Jean-Luc Vachiéry, Gianfranco Parati, Luigi P. Badano, Denisa Muraru

**Affiliations:** ^1^Department of Cardiology, Ospedale San Luca, Istituto Auxologico Italiano IRCCS, Milan, Italy; ^2^Department of Management, Information and Production Engineering, University of Bergamo, Bergamo, Italy; ^3^Department of Medicine and Surgery, University of Milano-Bicocca, Milan, Italy; ^4^Biostatistic Unit, IRCCS Istituto Auxologico Italiano, Milan, Italy; ^5^Department of Statistics and Quantitative Methods, Università di Milano-Bicocca, Milan, Italy; ^6^Department of Cardiology, Hôpital Académique Erasme, Cliniques Universitaires de Bruxelles, Brussels, Belgium

**Keywords:** right heart catheterization, tricuspid regurgitation, heart failure with preserved ejection faction, exercise, oxygen consumption, hemodynamics

## Abstract

**Background:**

Both secondary tricuspid regurgitation (STR) and heart failure with preserved ejection fraction (HFpEF) are relevant public health problems in the elderly population, presenting with potential overlaps and sharing similar risk factors. However, the impact of severe STR on hemodynamics and cardiorespiratory adaptation to exercise in HFpEF remains to be clarified.

**Aim:**

To explore the impact of STR on exercise hemodynamics and cardiorespiratory adaptation in HFpEF.

**Methods:**

We analyzed invasive hemodynamics and gas-exchange data obtained at rest and during exercise from HFpEF patients with severe STR (HFpEF-STR), compared with 1:1 age-, sex-, and body mass index (BMI)- matched HFpEF patients with mild or no STR (HFpEF-controls).

**Results:**

Twelve HFpEF with atrial-STR (mean age 72 years, 92% females, BMI 28 Kg/m^2^) and 12 HFpEF-controls patients were analyzed. HFpEF-STR had higher (*p* < 0.01) right atrial pressure than HFpEF-controls both at rest (10 ± 1 vs. 5 ± 1 mmHg) and during exercise (23 ± 2 vs. 14 ± 2 mmHg). Despite higher pulmonary artery wedge pressure (PAWP) at rest in HFpEF-STR than in HFpEF-controls (17 ± 2 vs. 11 ± 2, *p* = 0.04), PAWP at peak exercise was no more different (28 ± 2 vs. 29 ± 2). Left ventricular transmural pressure and cardiac output (CO) increased less in HFpEF-STR than in HFpEF-controls (interaction *p*-value < 0.05). This latter was due to lower stroke volume (SV) values both at rest (48 ± 9 vs. 77 ± 9 mL, *p* < 0.05) and at peak exercise (54 ± 10 vs. 93 ± 10 mL, *p* < 0.05). Despite these differences, the two groups of patients laid on the same oxygen consumption isophlets because of the increased peripheral oxygen extraction in HFpEF-STR (*p* < 0.01). We found an inverse relationship between pulmonary vascular resistance and SV, both at rest and at peak exercise (*R*^2^ = 0.12 and 0.19, respectively).

**Conclusions:**

Severe STR complicating HFpEF impairs SV and CO reserve, leading to pulmonary vascular de-recruitment and relative left heart underfilling, undermining the typical HFpEF pathophysiology.

## Background

The development of transcatheter interventions might enlarge the number of candidates to tricuspid regurgitation (TR) repair, including elderly patients with several comorbities and secondary TR (STR) (1–4). Among patients with STR, a high prevalence of patients with heart failure with preserved ejection fraction (HFpEF) might be expected: (i) aging represents a strong risk factor both HFpEF (5) and STR (2, 6); (ii) atrial fibrillation is inextricably linked to HFpEF (7) and to atrial-STR (6, 8–13); (iii) long-standing HFpEF may induce pulmonary hypertension (14), which might predispose to ventricular-STR (12, 13, 15). Thus, the clinical manifestations of HFpEF and severe STR may overlap and “confound” each other. Additionally, the net hemodynamic effect of developing STR in patients with an underlying hemodynamic abnormality, such as HFpEF, has not been clarified. Nonetheless, such information might be useful to figure out symptoms' pathophysiology of HFpEF-STR more clearly. In turn, this may allow to better tailor specific interventions in this population that it is expected to grow exponentially in the future, due to the progressive aging of the general population. Thus, the aim of this study was to explore the hemodynamic alterations related to STR in patients with hemodynamically-proven HFpEF by combining right heart catheterization with cardiopulmonary exercise test.

## Methods

This study was approved by the Ethics Committee of the Istituto Auxologico Italiano (protocol n 2020_04_21_03 approved on April 21st, 2020). All patients signed a written informed consent to allow the use of their clinical data for research purposes.

We analyzed the cohort of patients who underwent an elective, clinically indicated cardiac catheterization at rest and during exercise at Istituto Auxologico Italiano between January 2018 and January 2022.

Cases were the patients with HFpEF and severe STR (HFpEF-STR). HFpEF was defined based on the presence of signs and/or symptoms of heart failure, a left ventricular (LV) ejection fraction (EF) >50%, associated with invasive hemodynamic demonstration of HFpEF. Invasive diagnosis of HFpEF was established in patients with an end-expiratory pulmonary artery wedge pressure (PAWP) at peak exercise equal or higher than 25 mmHg, and/or PAWP/ cardiac output (CO) slope higher than 2 mmHg/L/min) (16–18). From 74 patients with HFpEF without severe TR, we selected an equal number of controls for an individual matching to cases (ratio 1:1) considering sex-, age- (±5 years), and body mass index- (±2 Kg/m^2^).

We excluded patients with LV EF < 50%, secondary forms of HFpEF (cardiomyopathy, infiltrative diseases, pericardial constriction), more than mild left-sided valvular heart disease, congenital heart disease, pulmonary vascular diseases (pulmonary arterial hypertension, chronic thromboembolic pulmonary hypertension), pulmonary hypertension due to lung disease and/or hypoxia, severe comorbidities. Additionally, we excluded patients with congenital or acquired primary TR, cardiac implanted electronic device-related TR, and previous tricuspid surgery. Among the cases, we distinguished between atrial- and ventricular-STR, based on the detection of invasive pulmonary artery systolic pressure lower or higher than 50 mmHg (15).

### Echocardiography

Echocardiography was performed according to current recommendations of the European Association of Cardiovascular Imaging and the American Society of Echocardiography by experienced cardiologists on the same day of the right heart catheterization (19), using a Vivid E9/E95 scanners (GE Vingmed, Horten, Norway). LV and left atrial (LA) volumes were measured using biplane disk summation algorithm on dedicated 4-chamber and 2-chamber apical views, taking care to avoid chamber foreshortening. Right atrial (RA) volume was measured in a right ventricle (RV) -focused 4-chamber view. Echocardiographic evaluation of STR severity was based on an integrative approach considering multiple qualitative and quantitative parameters (6, 20, 21). Due to the retrospective nature of the study, RV dimensions were qualitatively estimated in all patients.

### Right heart catheterization and cardiopulmonary exercise test

Patients were studied on optimized medical therapy and in euvolemic state, in non-fasting state, without sedation, and in supine position. They wore a non-rebreathing Hans-Rudolph mask connected to the V-MAX metabolic cart (Vmax SensorMedics 2200, Yorba Linda, CA, USA) to directly measure gas-exchange data and ventilation (17). A 7-F fluid-filled Swan-Ganz catheter was placed in the pulmonary artery through the right internal jugular vein under fluoroscopic guidance. Proper pulmonary artery wedge positioning was confirmed by the appearance of a typical PAWP trace as well as by an oxygen saturation >94% sampled at the tip of the catheter. The right radial artery was cannulated with the Seldinger technique. The transducer was zeroed at the midthoracic line, halfway between the anterior sternum and the bed surface. Hemodynamic measurements were performed at rest, after 1 min of passive leg raise (feet on the pedals), and during the last minute of each step of a symptom-limited, maximal exercise test (18). Two milliliters of blood were sampled at the same time from the tip of the Swan-Ganz catheter and from the radial artery for blood gases analysis. The increment in workload was personalized in order to obtain at least three steps of exercise before exhaustion. Subjects were encouraged to exercise up to their maximal volitional effort. Pulmonary artery pressure, PAWP and RA pressure (RAP) were reported as an average of several respiratory cycles (18). Hemodynamic data reflect the agreement of two readers who visually reviewed all pressure traces. CO was calculated by direct Fick method, solving the oxygen consumption (VO_2_) equation as follows: CO = VO_2_/C(a-v)O_2_, where C(a-v)O_2_ is the oxygen arteriovenous difference. Furthermore, to evaluate the relative contribution of the elements of the Fick equation to exercise capacity, we plotted CO as a function of C(a-v)O_2_ on which we represented VO_2_-isophlets (22).

LV trans-mural pressure (LVTMP), as a measure of LV preload, independent of right heart filling and pericardial restraint, was calculated as PAWP–RAP (23). We plotted the relationship between pulmonary vascular resistance (PVR) and stroke volume (SV) to explore whether low anterograde SV may be linked to pulmonary vascular derecruitment and higher than normal PVR (24).

Key ergospirometric measurements included standard breath-by-breath cardiorespiratory and breathing pattern parameters. Peak VO_2_ was measured as the highest 30-s value obtained at the end of the effort. The slope of the relationship between minute ventilation and carbon dioxide production (VE/VCO_2_ slope) was calculated over the linear component of VE vs. VCO_2_ (25).

### Statistics

Continuous variables are reported as mean and standard deviation (SD) or median and interquartile range if the data did not follow a normal distribution. The categorical variables are shown as absolute frequencies and proportions. For data at rest, unpaired *T*-test (or Wilcoxon signed rank sum test in case of non-normal distribution) was applied to compare the continuous variables between HFpEF-STR and HFpEF-controls, while Chi-square test (or Fisher test) was used to compare the categorical variables. For each hemodynamic variable measured during exercise, an ANOVA model for repeated measures was fitted, considering an unstructured variance-covariance matrix to take into account the correlation among measurements on the same subjects. The included covariates in each model were group (HFpEF-STR or HFpEF-controls), time (Rest, Leg Raise and Peak) and their interaction. The statistical significance of interaction term suggested a different trend of the hemodynamic variables between groups. Moreover, we tested the least square means (LS-means) differences among groups at each time by means of unpaired *t*-test applying the False Discovery Rate (FDR) approach to control the inflation of the type I error.

The relationship between continuous hemodynamic variables at specific time-point was investigated by means quadratic B-spline with 1 knot. The goodness of fit of the model was estimated by means of R2. This value, ranging from 0 to 1, represents the proportion of total variance of an independent variable explained by a dependent variable.

All analyses were performed using SAS version 9.4 software (SAS Institute, Cary, NC, USA). Statistical significance was set at the 0.05 level. All *P*-values were two-sided.

## Results

### Clinical characteristics

**Figure 1:**
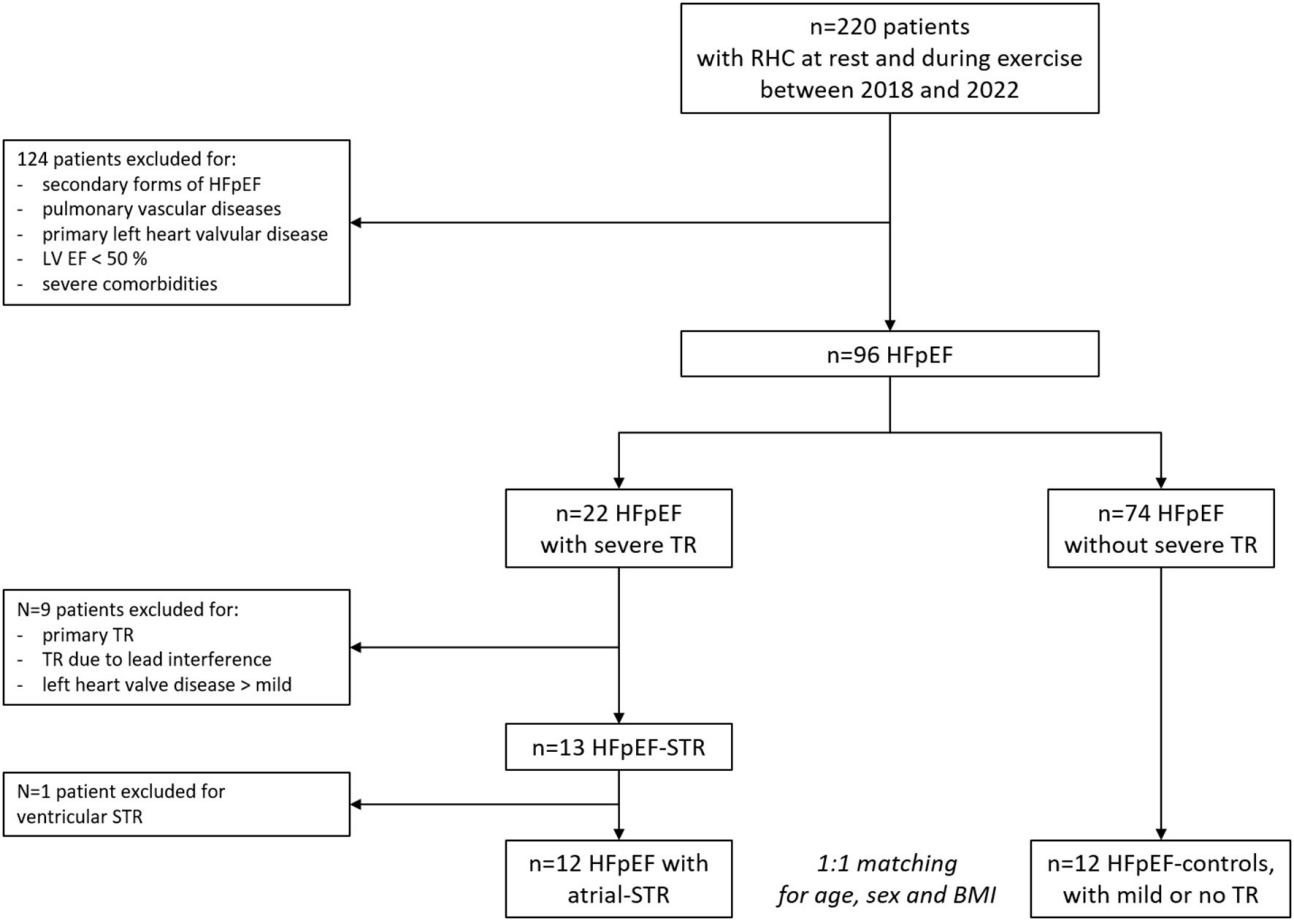
Patients' selection flow-chart. BMI, body mass index; HFpEF, heart failure with preserved ejection fraction; LV EF, left ventricular ejection fraction; RHC, right heart catheterization; STR, secondary tricuspid regurgitation; TR, tricuspid regurgitation.

Patients' selection flowchart is depicted in Figure 1. HFpEF-STR represented 14% of our HFpEF population who had undergone exercise right heart catheterization during the study period. All HFpEF-STR patients but one (who had a typical HFpEF profile, but presented with a systolic PAP >50 mmHg) fulfilled the criteria for atrial-STR. In order to provide results on a more homogeneous patients' phenotype, we excluded from the analysis the patient that presented with a ventricular-STR. Indeed, the analyses focused on HFpEF with atrial-STR as compared with HFpEF-controls.

The clinical characteristics of our study groups are summarized in Table 1. They represented a quite typical elderly, overweight and predominantly female HFpEF population. Of note, 83% of patients with HFpEF-STR had permanent atrial fibrillation, as compared with only 8% of permanent atrial fibrillation in HFpEF-controls (*p* < 0.01). The rhythm at the time of right heart catheterization was atrial fibrillation in 83% of HFpEF-STR and 8% of HFpEF-controls. Among the other cardiovascular comorbidities, arterial hypertension was the most common affecting 75% of HFpEF-controls and 83% of HFpEF-STR patients (*p* = 1.000).

**Table 1:** Clinical characteristics of the study cohort.

	**Whole cohort (*n* = 24)**	**HFpEF-controls (*n* = 12)**	**HFpEF-STR (*n* = 12)**	***P*-value**
**Anthropometrics and demographics**				
Age, years	72 ± 5	72 ± 5	72 ± 5	0.867^†^
Female sex, *n* (%)	22 (92%)	11 (92%)	11 (92%)	1.000^¥^
BMI, Kg/m^2^	28 ± 5	27 ± 4	28 ± 5	0.768^†^
**Comorbidities**				
Arterial hypertension, *n* (%)	19 (79%)	9 (75%)	10 (83%)	1.000^¥^
Diabetes mellitus, *n* (%)	1 (4%)	0 (0%)	1 (8%)	1.000^¥^
Coronary artery disease, *n* (%)	3 (13%)	2 (17%)	1 (8%)	1.000^¥^
Paroxysmal/permanent AFib, *n* (%)	12 (50%)	2 (17%)	10 (83%)	0.001^||^
Pace-maker, *n* (%)	2 (8)	1 (8)	1 (8)	1.000^¥^
Previous cardiac surgery, *n* (%)	2 (8)	1 (8)	1 (8)	1.000^¥^
**Treatment**				
Furosemide, *n* (%)	16 (68%)	7 (58%)	9 (75%)	0.667^¥^
Spironolactone, *n* (%)	8 (33%)	4 (33%)	4 (33%)	1.000^¥^
Hydrochlorothiazide, *n* (%)	3 (13%)	2 (17%)	1 (8%)	1.000^¥^
ACE-I/ARB, *n* (%)	14 (58%)	7 (58%)	7 (58%)	1.000^||^
Beta-blocker, *n* (%)	17 (71%)	6 (50%)	11 (92%)	0.069^¥^
Oral anticoagulant, *n* (%)	12 (50%)	2 (17%)	10 (83%)	0.001^||^
**Symptoms**				
NYHA class III-IV, *n* (%)	15 (63%)	9 (75%)	6 (50%)	0.400^||^
Peripheral edema, *n* (%)	10 (42%)	4 (33%)	6 (50%)	0.408^||^
JVD, *n* (%)	3 (13%)	0 (0%)	3 (25%)	0.217^¥^
**Blood tests**				
Hemoglobin, g /dL	12.7 ± 1.4	12.7 ± 1.4	12.7 ± 1.5	0.933^†^
BNP	251 ± 185	220 ± 202	289 ± 168	0.451^†^
eGFR, mL/min/1.73 m^2^	57 ± 20	61 ± 23	54 ± 17	0.457^†^
AST, mg/dL	23 [21–30]	22.5 [19-25.5]	24 [21–30]	0.204^‡^
ALT, mg/dL	17.5 [14–26]	17 [13–19]	24.5 [18–40]	0.013^‡^
**Echocardiography**				
LVEF, %	63 ± 5	63 ± 5	63 ± 5	0.980^†^
LVEDVI, mL	47 ± 9	50 ± 8	44 ± 9	0.166^†^
RV dilation, *n* (%)	8 (38%)	1 (11%)	7 (58%)	0.067^¥^
LAVI, mL/m^2^	44 ± 13	36 ± 10	50 ± 12	0.007^†^
RAVI, mL/m^2^	37 ± 18	25 ± 10	46 ± 18	0.003^†^
**Cardiopulmonary exercise test**				
VO_2_, % of predicted	74 ± 18	77 ± 21	71 ± 15	0.446^†^
VO_2_, mL/Kg/min	13 ± 4	13 ± 3	12 ± 4	0.690^†^
CO/VO_2_ slope	4.7 ± 1.8	5.5 ± 1.7	3.9 ± 1.5	0.024^†^
VE/VCO_2_ slope	33 ± 5	35 ± 6	32 ± 5	0.327^†^

Continuous data are shown as mean ± standard deviation or median [interquartile range]. Categorical data are shown as number (percentage).

AFib, atrial fibrillation; ALT, alanine aminotransferase; AST, aspartate aminotransferase; BMI, body mass index; BNP, brain natriuretic peptide; CO, cardiac output; eGFR, estimated glomerular filtration rate; JVD, jugular vein distension; LAVI, left atrial volume index; LVEDVI, left ventricular end-diastolic volume index; LVEF, left ventricular ejection fraction; RAVI, right atrial volume index; RV, right ventricle; VCO_2_, carbon dioxide production; VE, minute ventilation; VO_2_, oxygen consumption.

^†^*T*-test;^‡^Wilcoxon test; ^||^Chi-square test; ^¥^Fisher test.

Representation of HF signs and symptoms did not significantly differ between HFpEF-STR and HFpEF-controls (Table 1). Brain natriuretic peptide values were slightly elevated but similar between the groups. Most of the blood test data reflecting secondary organ damage (renal, hepatic) showed similar values in HFpEF-STR as compared with HFpEF-controls. Only alanine aminotransferease values resulted significantly higher in HFpEF-STR patients than in HFpEF-controls. Estimated glomerular filtration rate was lower than expected, especially in HFpEF-STR, but did not significantly differ between the two groups (Table 1).

LV geometry and function were similar between the two groups, while RV dilation was non-significantly more frequent in HFpEF-STR than in HFpEF-controls (58 vs. 11%, *p* = 0.067). As expected, because of the incidence of atrial fibrillation, patients with HFpEF-STR presented larger LA and RA volumes that were, respectively, 43 and 84% larger than in HFpEF-controls (respectively, *p* < 0.01). No patient with HFpEF-STR presented with leaflet tenting.

### Right heart catheterization and exercise capacity

Complete hemodynamic data are shown in Table 2 and Supplementary Table 1, while relative changes in hemodynamics from rest to feet on the pedals and between rest and peak are shown in Table 3.

**Table 2:** Hemodynamics at rest, with feet on the pedals and at peak exercise in the study cohort.

	**Rest**		**Leg raise**		**Peak**	
	**HFpEF-controls**	**HFpEF- STR**	**HFpEF-controls**	**HFpEF- STR**	**HFpEF-controls**	**HFpEF- STR**
HR, bpm	66 ± 4	81 ± 4^*^	68 ± 4	81 ± 4^*^	99 ± 6	120 ± 6^*^
SVI, mL/min/m^2^	43 ± 4	28 ± 4^*^	45 ± 4	32 ± 4^*^	52 ± 2	32 ± 5^*^
CI, mL/min/m^2^	2.8 ± 0.2	2.2 ± 0.2	2.9 ± 0.2	2.5 ± 0.2	4.9 ± 0.3	3.7 ± 0.3^*^
C(a-v)O_2_, mL/dL	4.2 ± 0.3	5.3 ± 0.3^**^	4.3 ± 0.3	6.0 ± 0.3^**^	11.2 ± 0.7	13.3 ± 0.7^*^
PVR, WU	1.5 ± 0.3	2.1 ± 0.3	1.3 ± 0.3	1.6 ± 0.3	1.1 ± 0.3	1.7 ± 0.3
mPAP, mmHg	18 ± 1	24 ± 1^*^	23 ± 2	27 ± 2	36 ± 2	39 ± 2
PAWP, mmHg	11 ± 2	17 ± 2^*^	16 ± 2	21 ± 2	28 ± 2	29 ± 2
LVMTP, mmHg	6 ± 1	7 ± 1	8 ± 1	8 ± 2	13 ± 2	7 ± 2^*^
RAP, mmHg	5 ± 1	10 ± 1^**^	8 ± 1	14 ± 1^**^	14 ± 2	23 ± 2^**^
RAP V wave, mmHg	6 ± 1	13 ± 1^***^	9 ± 2	18 ± 2^***^	17 ± 2	28 ± 2^***^
RAP/PAWP	0.45 ± 0.08	0.64 ± 0.08	0.52 ± 0.05	0.67 ± 0.05^*^	0.50 ± 0.06	0.79 ± 0.06^**^

CI, cardiac index; HFpEF, heart failure with preserved ejection fraction; HR, heart rate; LVMTP, left ventricular transmural pressure; mPAP, mean pulmonary artery pressure; PAWP, pulmonary artery wedge pressure; PVR, pulmonary vascular resistance; RAP, right atrial pressure; STR, secondary tricuspid regurgitation; SVI, stroke volume index. ^*^p < 0.05; ^**^p < 0.01; ^***^p < 0.001.

**Table 3:** Changes in hemodynamics between rest and feet on the pedals, and between rest and at peak exercise in the study cohort.

	**Delta feet on pedals—Rest**			**Delta peak exercise—Rest**		
	**HFpEF-controls**	**HFpEF-STR**	***P*** **-value**	**HFpEF-controls**	**HFpEF-STR**	***P*** **-value**
HR, bpm	2 ± 4	−0.3 ± 4	0.713	33 ± 4	39 ± 5	0.296
SVI, mL/min/m^2^	2 ± 2	4 ± 2	0.519	9 ± 2	4 ± 2	0.174
CI, mL/min/m^2^	0.2 ± 0.2	0.3 ± 0.2	0.669	2.2 ± 0.2	1.5 ± 0.2	0.036
C(a-v)O2, mL/dL	0.26 ± 0.47	0.72 ± 0.47	0.492	6.84 ± 0.47	7.69 ± 0.47	0.099
PVR, WU	−0.2 ± 0.2	−0.6 ± 0.2	0.286	−0.5 ± 0.2	−0.4 ± 0.2	0.924
mPAP, mmHg	5 ± 1	4 ± 1	0.562	18 ± 1	15 ± 1	0.167
PAWP, mmHg	5 ± 1	5 ± 1	0.731	17 ± 1	13 ± 1	0.017
LVMTP, mmHg	2 ± 1	1 ± 1	0.548	8 ± 1	0 ± 1	0.0001
RAP, mmHg	4 ± 1	4 ± 1	0.724	9 ± 1	13 ± 1	0.019
RAP V wave, mmHg	3 ± 1	5 ± 1	0.294	11 ± 1	15 ± 1	0.019

CI, cardiac index; HFpEF, heart failure with preserved ejection fraction; HR, heart rate; LVMTP, left ventricular transmural pressure; mPAP, mean pulmonary artery pressure; PAWP, pulmonary artery wedge pressure; PVR, pulmonary vascular resistance; RAP, right atrial pressure; STR, secondary tricuspid regurgitation; SVI, stroke volume index.

At rest, patients with HFpEF-STR had higher mean pulmonary artery pressure (24 ± 1 mmHg vs. 18 ± 1 mmHg, *p* = 0.006) and PAWP (17 ± 2 mmHg vs. 11 ± 2 mmHg, *p* = 0.006) than HFpEF-controls, consistent with mild post-capillary pulmonary hypertension (PH). PH, defined by a mean pulmonary artery pressure >20 mmHg, was present in 83% of HFpEF-STR and in 25% of HFpEF-controls. SV was lower in HFpEF-STR than in HFpEF-controls (28 ± 4 mL vs. 43 ± 4 mL/m^2^, *p* = 0.03), which was compensated by higher heart rate (81 ± 4 bpm vs. 66 ± 4 bpm, *p* = 0.022) to maintain cardiac index, which resulted on average at the lower limit of normal in HFpEF-STR (2.2 ± 0.2 L/min/m^2^ vs. 2.8 ± 0.2 L/min/m^2^, *p* = 0.147). PVR was slightly increased in HFpEF-STR patients but not significantly higher to that of HFpEF-controls (2.1 ± 0.3 WU vs. 1.5 ± 0.3 WU, *p* = 0.196). RAP was twice as high in HFpEF-STR than in HFpEF-controls (10 ± 1 mmHg vs. 5 ± 1 mmHg, *p* = 0.007), with tall V waves (13 ± 1 mmHg vs. 6 ± 1 mmHg, *p* < 0.001) and a high RAP/PAWP ratio (0.64 ± 0.08 vs. 0.45 ± 0.08, *p* = 0.079).

**Figure 2:**
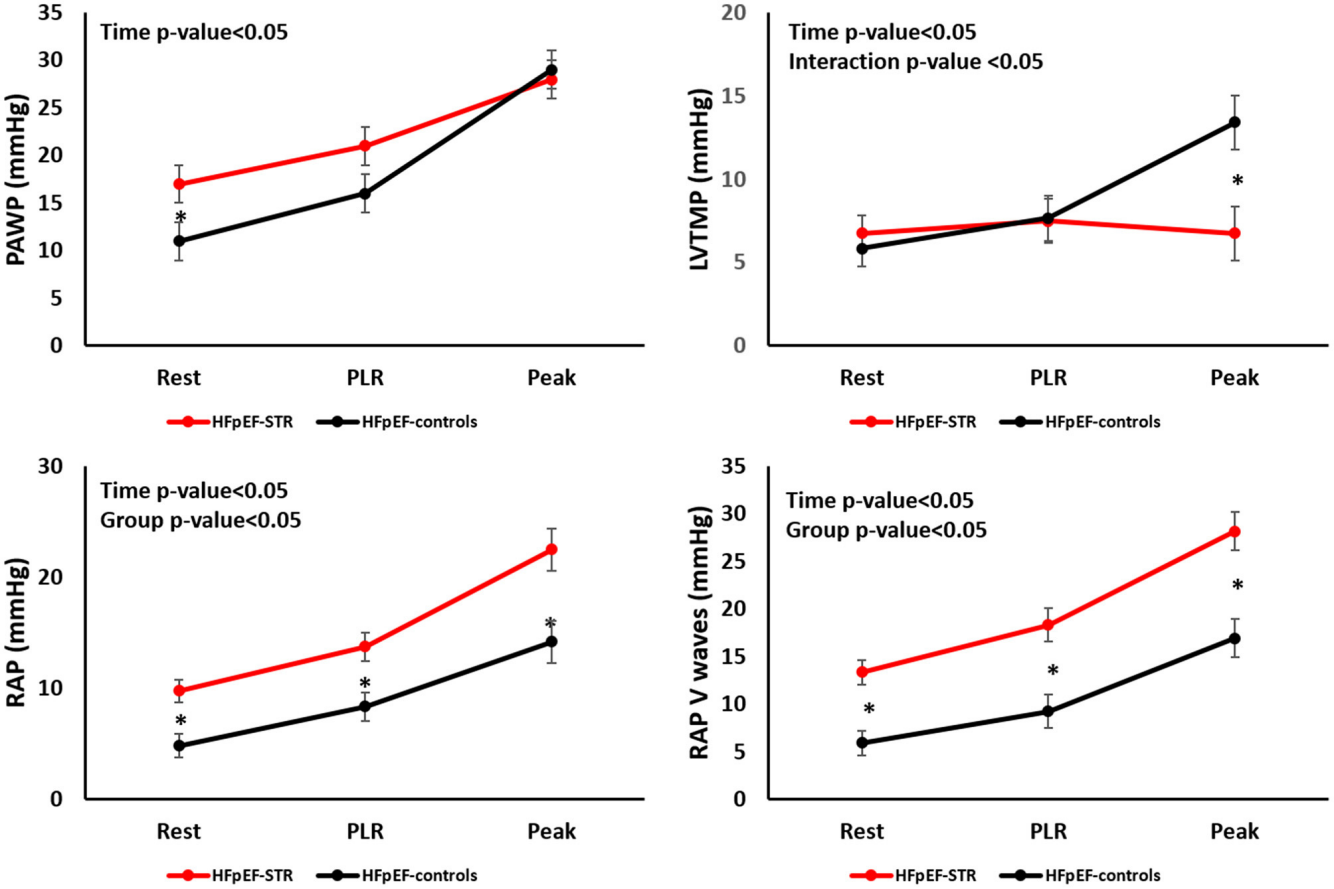
Evolution of left and right heart hemodynamics during exercise in our patients' population. HFpEF, heart failure with preserved ejection fraction; LVTMP, left ventricular transmural pressure; PAWP, pulmonary artery wedge pressure; PLR, passive leg raising; RAP, right atrial pressure; STR, secondary tricuspid regurgitation. *P < 0.05.

During exercise (Figure 2, Table 3, and Supplementary Table 1), PAWP increased less in HFpEF-STR patients than in HFpEF-controls. Indeed, despite higher PAWP in HFpEF-STR than in HFpEF-controls at rest, at peak exercise PAWP was not different in the two groups (28 ± 2 vs. 29 ± 2, *p* = 0.584). RAP V waves displayed an opposite behavior, increasing more in HFpEF-STR patients than in HFpEF-controls (interaction *p*-value = 0.06), with persistently higher RAP and RAP/PAWP ratio in HFpEF-STR patients than in HFpEF-controls (group *p*-value < 0.01). Accordingly, LVTMP increased only in HFpEF-controls during exercise (interaction *p*-value = 0.006), coherent with LV underfilling in HFpEF-STR patients.

**Figure 3:**
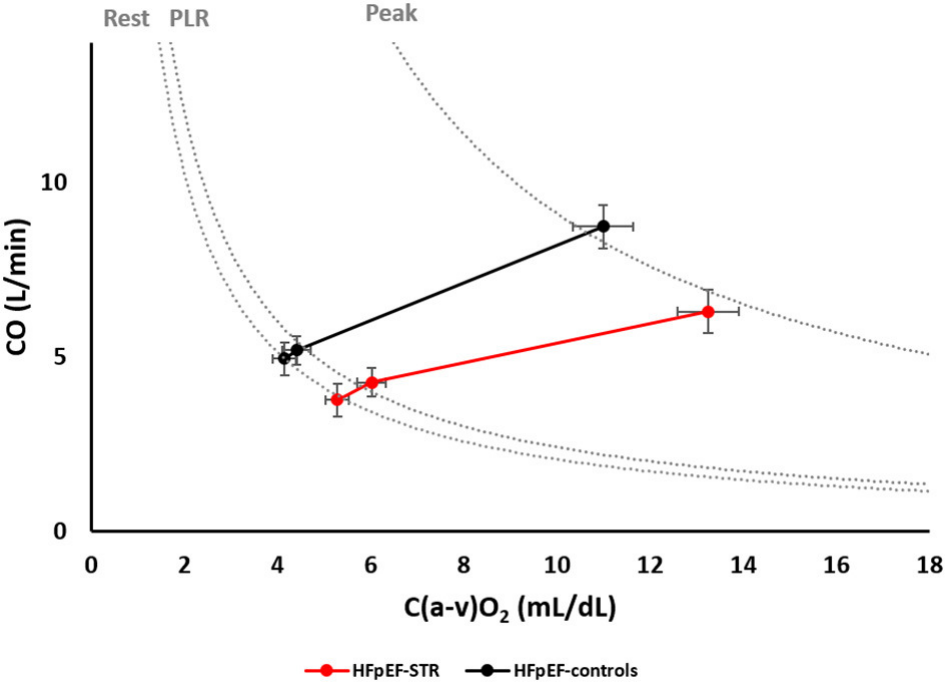
Relative weight of cardiac output and arteriovenous oxygen difference in determining oxygen consumption at rest, during passive leg raising and at peak exercise. Dotted lines represent oxygen consumption isophlets, i.e., cardiac output and arteriovenous oxygen difference coordinates whose product is a given oxygen consumption, at rest, during passive leg raising, and at peak exercise. C(a-v)O_2_, arteriovenous oxygen difference; CO, cardiac output; HFpEF, heart failure with preserved ejection fraction; PLR, passive leg raising; STR, secondary tricuspid regurgitation.

Exercise capacity, as evaluated through peak VO_2_, was not different at peak exercise between HFpEF-STR and HFpEF-controls, who reached the 71 and 77% of predicted values, respectively (*p* = 0.446), as shown in Table 1. However, the determinants of VO_2_ (Table 2, Supplementary Table 1, and Figure 3) behaved differently in the two groups, with a lower increase in CO and a higher C(a-v)O_2_ in HFpEF-STR patients as compared with HFpEF-controls (group *p*-value < 0.05 for both variables). In particular, HFpEF-STR and HFpEF-controls roughly lay roughly on the same VO_2_ isophlets at each step of exercise, with HFpEF-STR rightward and downward shifted (Figure 3). Accordingly, the CO/VO_2_ slope, as a measure of CO reserve, was lower in HFpEF-STR patients than in HFpEF-controls (3.9 vs. 5.5, *p* = 0.024). Exercise hyperventilation (VE/VCO2 slope) did not differ between HFpEF-STR and HFpEF-controls.

The SV was lower in HFpEF-STR than in HFpEF-controls (group *p*-value = 0.018). In particular, at peak exercise, it resulted 40 mL lower in HFpEF-STR (*p* = 0.03). The rate of increase of SV in the two groups however did not significantly differ (Table 3). Similarly, PVR similarly decreased by 0.4–0.5 WU in both groups during exercise. SV and PVR were inversely correlated both at rest and peak, with higher PVR at lower SV (Supplementary Figure 1). However, the low *R*^2^ values (0.12 and 0.19 for rest and peak timepoint, respectively) suggested high heterogenity in the relationship.

## Discussion

**Figure 4:**
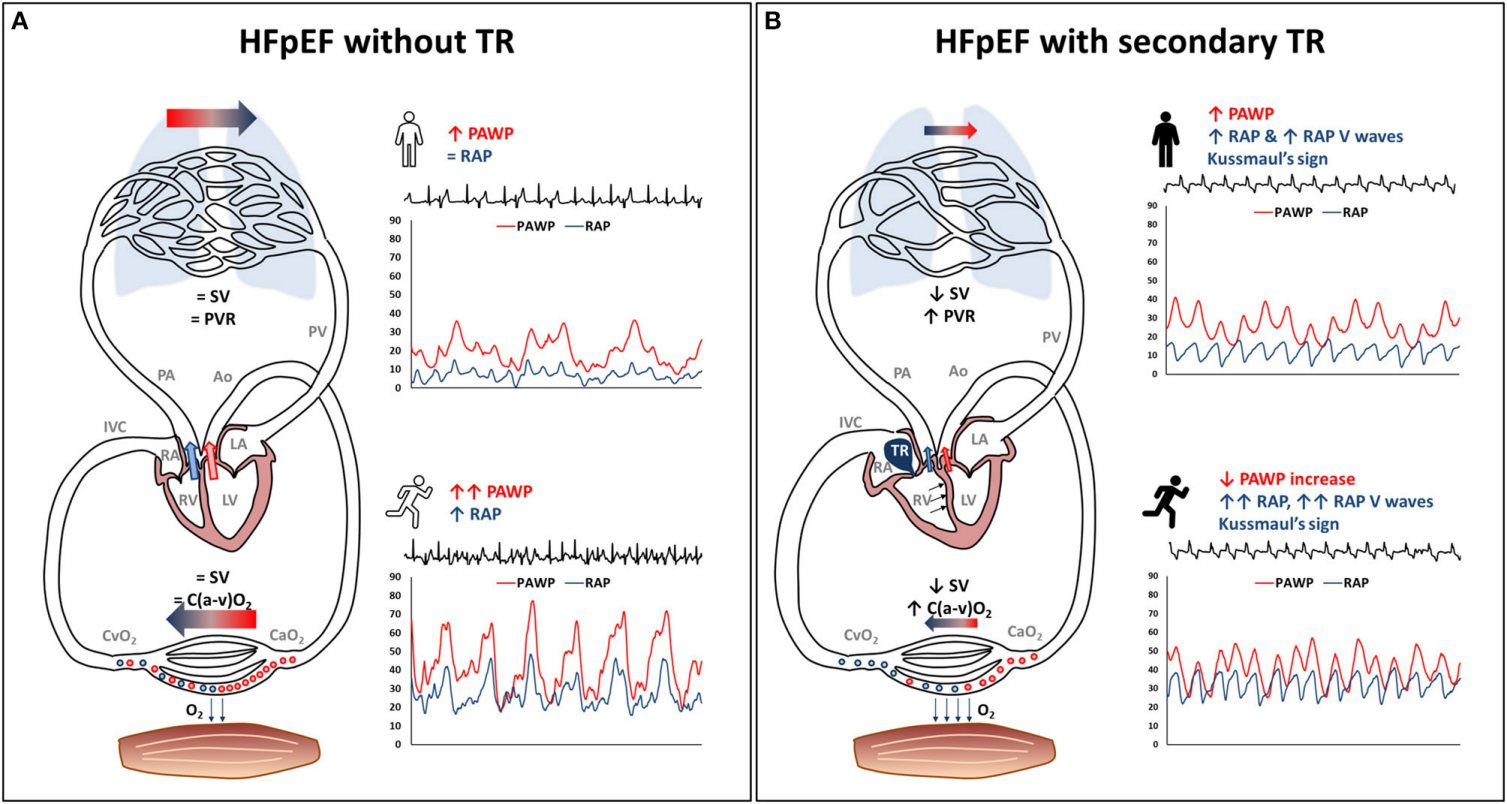
Exercise pathophysiology of secondary tricuspid regurgitation (STR) in heart failure with preserved ejection fraction (HFpEF). The typical HFpEF patient without STR (panel on the left) may present with **(A)** high pulmonary artery wedge pressure, either at rest or during exercise, with a steep pulmonary artery wedge pressure rise, and mildly increased right atrial pressure. **(B)** substantially normal stroke volume, pulmonary vascular resistance and peripheral oxygen extraction. This HFpEF pathophysiology is altered in the presence of STR (panel on the right), being characterized by: (i) high right atrial pressure that lacks inspiratory decrease or may present with overt Kussmaul's sign, tall V waves in the right atrium, right atrial enlargement and venous congestion; (ii) reduced forward stroke volume with pulmonary vascular derecruitment (increasing pulmonary vascular resistance), left ventricular underfilling, flatter pulmonary artery wedge pressure rise during exercise, and high ratio between right atrial pressure and pulmonary artery wedge pressure; (iii) higher reliance upon peripheral oxygen extraction to exercise despite low stroke volumeAo, aorta; C(a-v)O2, arteriovenous oxygen difference; CaO2, arterial oxygen content; CvO2, venous oxygen content; IVC, inferior vena cava; LA, left atrium; LV, left ventricle; O_2_, oxygen; PA, pulmonary artery; PAWP, pulmonary artery wedge pressure; PV, pulmonary vein; PVR, pulmonary vascular resistance; RAP, right atrial pressure; SV, stroke volume; TR, tricuspid regurgitation.

Our study highlights the hemodynamic impact of developing STR in a cohort of patients with HFpEF. Indeed, in patients with HFpEF, STR was associated with (i) marked RA dilation and RA hypertension, both at rest and during exercise; (ii) reduced SV and CO reserve, with patients relying on heart rate and on peripheral compensation (O_2_ extraction) to maintain exercise performance; (iii) pulmonary vascular derecruitment and LV underfilling, leading to lower than expected increase in left heart filling pressure during exercise (Figure 4).

In our relatively small cohort of highly-selected and hemodynamically-proven HFpEF patients who underwent a clinically-indicated right heart catheterization at rest and during exercise, STR prevalence was not irrelevant (14%). The etiology of severe STR was atrial-functional in the great majority of these patients, on whom we decided to focus our analyses. Of note, 83% of patients with atrial-STR had a hemodynamic diagnosis of PH at rest according to the new definition (14), even though the degree of mean pulmonary artery pressure elevation was only mild, and no patient presented with leaflet tenting. Indeed, the definition of atrial-STR (previously known as isolated TR) (6, 8–15) relies on exclusion of other more commonly known secondary causes of TR and on an imprecise echocardiographic surrogate to exclude severe PH, i.e., a systolic pulmonary artery pressure < 50 mmHg (15). This distinction between ventricular-STR and atrial-STR may be better refined in the future, taking advantage of 3D reconstruction of the RV, the RA and of the tricuspid annulus, and considering the relative contribution of each of these elements in the pathogenesis of TR, as well as more precise measures of RV afterload.

Coherent with the non-negligible prevalence of atrial-STR in our hemodynamically-proven HFpEF population, both atrial-STR and HFpEF share common denominators, such as aging and atrial fibrillation. First, the prevalence of STR increases with age (26). Similarly, HFpEF is a disease of cardiovascular aging (27), which promotes diastolic stiffening of the LV and LA myopathy (28). Second, atrial fibrillation is associated with RA enlargement, negative remodeling of the tricuspid annular-valvular complex, and development of atrial-functional STR (8, 9, 11–13, 29–32). On the other hand, it has been proven that HFpEF diagnosis is a risk factor for the development or the progression of atrial fibrillation (28) and that atrial fibrillation is a strong clue for HFpEF diagnosis (33). Additionally, atrial fibrillation plays a role in HFpEF progression: its persistence may be associated over time with adverse bi-atrial remodeling, volume expansion, development of STR and right ventricular dysfunction (5, 10, 34, 35).

RAP, although often within normal limits in early-stage HFpEF, is generally 2-fold higher in this population than in healthy controls (16). STR causes an additional impact on the right heart of HFpEF patients, elevating RAP by another factor 2 in our HFpEF-STR. Despite this marked RAP elevation both at rest and during exercise, symptoms and peak VO_2_ did not relevantly differ between HFpEF-STR patients and HFpEF controls. Nonetheless, HFpEF-STR systematically presented with lower SV, and had to rely more on heart rate and peripheral O_2_ extraction [despite this latter being slightly lower than normal (36), pointing to the additional component of peripheral limitation in HFpEF] to maintain an exercise performance similar to HFpEF-controls. This highlights the importance of avoid strict rate-control strategies in HFpEF patients with atrial-STR (37). Additionally, our results suggest that VO_2_ at peak exercise might not be an optimal surrogate for STR severity, since HFpEF patients showed an ability to compensate for low CO by increasing peripheral extraction and, consequently, that interventions aimed at reducing STR severity might not necessarily improve peak VO_2_ despite improving symptoms, nonetheless reducing muscular fatigue. Furthermore, low SV was associated with pulmonary vascular de-recruitment, that can lead to spuriously increased PVR (24), not necessarily reflecting pulmonary vascular remodeling. Indeed, the rate of decrease in PVR during exercise was similar in the two groups, with HFpEF-STR set at higher PVR values because of low SV. Thus, slightly increased PVR at rest should not necessarily contraindicate transcatheter interventions for the correction of STR (38), albeit potentially indicating a worse patients' profile (39, 40). Finally, low SV together with the marked increase in RAP probably contributed to LV underfilling in HFpEF-STR patients by reducing LVTMP (23). This apparently counterintuitive finding, that volume-expanded HFpEF-STR patients had actually lower than expected rise in PAWP during exercise, might have clinical implications. Indeed, one might guess that STR may “protect” HFpEF-STR patients from pulmonary congestion, at the expense of reduced forward flow, even though pulmonary congestion might be favored by impaired lymphatic drainage associated with RA hypertension (41). After STR correction, unimpeded LV filling as a result of increased SV, may be associated with a sharp rise in left heart filling pressure with more overt pulmonary congestion, potentially fading the net clinical benefit of tricuspid valve repair.

### Study limitations

This is a small, single-center retrospective study, conducted on a highly selected patients' population. To investigate the effect of STR on HFpEF pathophysiology, we sought to limit potential confounders by applying a 1:1 matching for age, sex, and body mass index, as well as rigorously identifying patients with HFpEF using exercise invasive hemodynamics. The careful and precise hemodynamic evaluation we performed, which could provide significant and physiologically-meaningful results, might at least partially compensate for the small sample size, albeit we cannot exclude the possibility of a type II error. However, we could not control for atrial fibrillation, which was expectedly more frequent in HFpEF-STR than in HFpEF-controls, and that might have contributed to some of the above-mentioned hemodynamic differences between these two population, which may eventually represent two extremes of the HFpEF progression (41). Additionally, the limited sample size of our well-characterized population might have hindered to highlight significant differences in some clinical variables between HFpEF-STR and HFpEF-controls. Due to the retrospective nature of the study, patients had only qualitative assessment of RV geometry and function, while three-dimensional evaluation would have been desirable for a better understanding of this complex chamber. Moreover, the use of exercise-stress echocardiography would have improved our understanding of hemodynamic behaviors, especially in terms of ventricular interdependence (42).

## Conclusion

Occurrence of atrial-STR in patients with HFpEF is associated with atrial fibrillation. The hemodynamic characteristics at rest and during exercise of HFpEF-STR patients are consistent with afterload-independent right heart failure. Due to low SV, HFpEF-STR have to rely more on heart rate and peripheral O_2_ extraction to maintain exercise capacity. Pulmonary vascular resistance in HFpEF-STR may be slightly increased due to pulmonary vascular derecruitment. LV underfilling may lead to lower than expected PAWP. Understanding this peculiar pathophysiology may be relevant in the perspective of transcatheter correction of STR in HFpEF patients.

## Data availability statement

The raw data supporting the conclusions of this article will be made available by the authors, without undue reservation.

## Ethics statement

This study was approved by the Ethics Committee of the Istituto Auxologico Italiano (protocol n 2020_04_21_03 approved on April 21st, 2020). All patients signed a written informed consent to allow the use of their clinical data for research purposes.

## Author contributions

CB contributed for conceptualization, data curation, investigation, methodology, visualization, and writing the original draft. SC contributed for conceptualization, data curation, investigation, methodology, supervision, and visualization and writing the original draft. GC contributed to data curation, investigation, methodology, and writing—review and editing. DS contributed for data analysis and writing—review and editing. AZ contributed for data analysis and writing—review and editing. CD contributed for supervision and writing—review and editing. MG, FH, MT, NR, and FP contributed to writing—review and editing. GPe contributed to supervision and writing—review and editing. J-LV contributed to conceptualization, supervision, and writing—review and editing. GPa contributed to supervision and writing—review and editing. LB and DM contributed to conceptualization, supervision, and writing—review and editing. All authors contributed to the article and approved the submitted version.
