# Supplementary material for: Impact of severe secondary tricuspid regurgitation on rest and exercise hemodynamics of patients with heart failure and a preserved left ventricular ejection fraction
Source: Front Cardiovasc Med. 2023 Mar 1;10:1061118. doi: 10.3389/fcvm.2023.1061118 (PMC10014840; doi:10.3389/fcvm.2023.1061118)
Supplement: Supplementary file 1 [file Data_Sheet_1.docx]

**Online data supplement**

**e-Table 1. ANOVA model for repeated measures for hemodynamic variables.** Included covariates in each model were group (HFpEF-STR or HFpEF-controls), time (Rest, Leg Raise and Peak) and their interaction.

|  | **Group P-value** | **Time p-value** | **Interaction P-value** |
| --- | --- | --- | --- |
| **HR, bpm** | 0.006 | <0.001 | 0.337 |
| **SVI, mL/min/m^2^** | 0.012 | <0.001 | 0.260 |
| **CI, mL/min/m^2^** | 0.033 | <0.001 | 0.107 |
| **C(a-v)O_2_, mL/dL** | 0.003 | <0.001 | 0.063 |
| **PVR, WU** | 0.225 | 0.014 | 0.413 |
| **mPAP, mmHg** | 0.031 | <0.001 | 0.430 |
| **PAWP, mmHg** | 0.107 | <0.001 | 0.111 |
| **LVMTP, mmHg** | 0.241 | 0.002 | 0.006 |
| **RAP, mmHg** | 0.003 | <0.001 | 0.158 |
| **RAP V waves, mmHg** | <0.001 | <0.001 | 0.063 |
| **RAP/PAWP** | 0.081 | 0.137 | 0.127 |

**Abbreviations.** CI, cardiac index; HFpEF, heart failure with preserved ejection fraction; HR, heart rate; LVMTP, left ventricular transmural pressure; mPAP, mean pulmonary artery pressure; PAWP, pulmonary artery wedge pressure; PVR, pulmonary vascular resistance; RAP, right atrial pressure; STR, secondary tricuspid regurgitation; SVI, stroke volume index.

**e-Figure 1. Inverse relationship between pulmonary vascular resistance and stroke volume in our patients’ cohort.** Panel A, resting conditions; panel B, peak exercise.


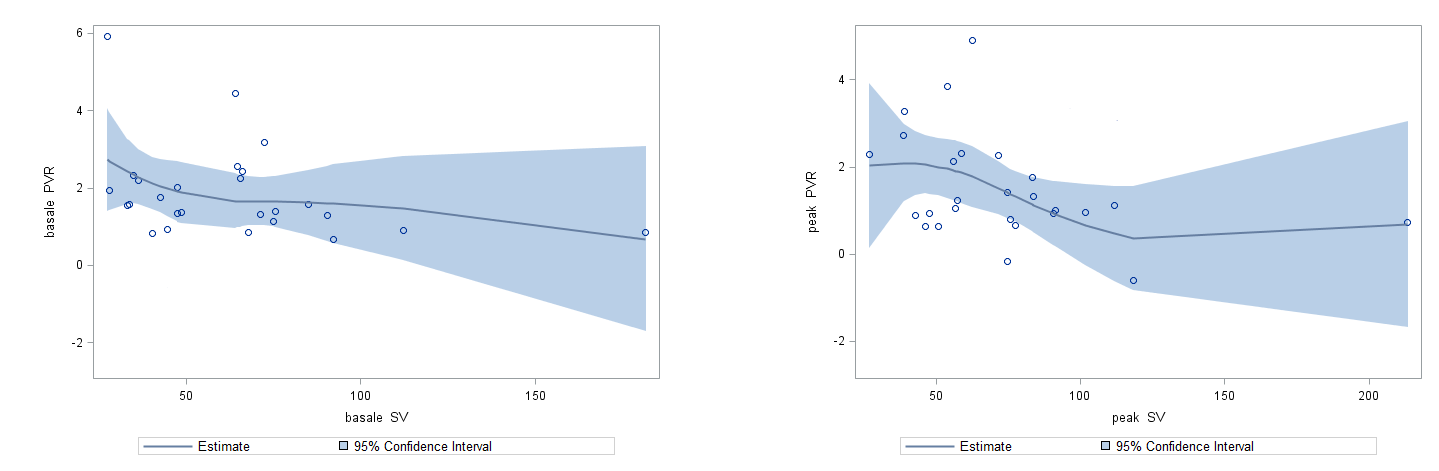


**Abbreviations.** HFpEF, heart failure with preserved ejection fraction; PVR, pulmonary vascular resistance; STR, secondary tricuspid regurgitation; SV, stroke volume.
